# Nano-TRAIL: a promising path to cancer therapy

**DOI:** 10.20517/cdr.2022.82

**Published:** 2023-02-01

**Authors:** Siri Chandana Gampa, Sireesha V. Garimella, SanthiLatha Pandrangi

**Affiliations:** ^1^Department of Biotechnology, Institute of Science, GITAM (Deemed to be University), Andhra Pradesh 530045, India.; ^2^Department of Biochemistry and Bioinformatics, Institute of Science, GITAM (Deemed to be University), Andhra Pradesh 530045, India.

**Keywords:** TRAIL, cancer cells, nanoparticles, apoptosis, nanomedicine

## Abstract

Tumor Necrosis Factor-Related Apoptosis-Inducing Ligand, also called apo-2 ligand (TRAIL/Apo-2L), is a cytokine that triggers apoptosis by binding to TRAIL-R1 (DR4) and TRAIL-R2 (DR5) death receptors. Apoptosis occurs through either the extrinsic or intrinsic pathway. The administration of recombinant human TRAIL (rhTRAIL) or TRAIL-receptor (TRAIL-R) agonists promotes apoptosis preferentially in cancerous cells over normal cells *in vitro*; this phenomenon has also been observed in clinical studies. The limited efficacy of rhTRAIL in clinical trials could be attributed to drug resistance, short half-life, targeted delivery issues, and off-target toxicities. Nanoparticles are excellent drug and gene delivery systems characterized by improved permeability and retention, increased stability and biocompatibility, and precision targeting. In this review, we discuss resistance mechanisms to TRAIL and methods to overcome TRAIL resistance by using nanoparticle-based formulations developed for the delivery of TRAIL peptides, TRAIL-R agonists, and TRAIL genes to cancer cells. We also discuss combinatorial approaches of chemotherapeutic drugs with TRAIL. These studies demonstrate TRAIL’s potential as an anticancer agent.

## INTRODUCTION

Cancer is the aberrant growth of cells capable of invading and metastasizing to other body parts. It is one of the leading causes of death, with approximately 10 million deaths expected globally by 2020, according to the World Health Organization. There are various treatment options, including surgery, chemotherapy, targeted therapy, radiation therapy, immunotherapy, and hormone therapy^[1]^. One branch of chemo/radiotherapy is targeted therapy that targets the tumor alone (for example, tumor vasculature or intracellular organelles, leaving the surrounding cells unaffected). This approach results in precise treatment of the tumor alone, reducing the drawbacks associated with the process^[2]^. Among the many targeted therapies is Tumor Necrosis Factor (TNF)-Related Apoptosis-Inducing Ligand, also known as Apo-2 ligand (TRAIL/Apo-2L).

TRAIL/Apo-2L is a type II transmembrane protein of the TNF superfamily of ligands. TRAIL binds to TRAIL-Receptor 1/Death Receptor 4 (TRAIL-R1/DR4), TRAIL-Receptor 2/Death Receptor 5 (TRAIL-R2/DR5), TRAIL-Receptor 3/Decoy Receptor 1 (TRAIL-R3/DcR1), and TRAIL-Receptor 4/Decoy Receptor 2 (TRAIL-R4/DcR2). It also binds to osteoprotegerin (OPG), which is soluble^[3,4]^. TRAIL selectively promotes apoptosis in cancer cells by the extrinsic (receptor-mediated) and intrinsic (mitochondria-mediated) pathways^[5-7]^. Apoptosis is a well-ordered and well-coordinated cellular process that maintains homeostasis in physiological and diseased conditions. In the extrinsic pathway, trimerization and subsequent receptor activation occur upon binding of TRAIL to its respective DR4 and DR5 death receptors, which leads to Death Inducing Signaling Complex (DISC) formation^[8]^. The intrinsic pathway includes mitochondrial outer membrane permeabilization (MOMP) and apoptosome formation^[9,10]^. Intrinsic and extrinsic mechanisms activate the executioner caspases (caspase-3/7), which cause DNA degradation and result in cell death. Here, we elaborate on the TRAIL-induced apoptotic pathways, resistance mechanisms exhibited by cancer cells to TRAIL therapy, and current approaches to overcome the resistance and utilize TRAIL as a potential cancer therapeutic agent.

### TRAIL and TRAIL receptors

**Figure 1:**
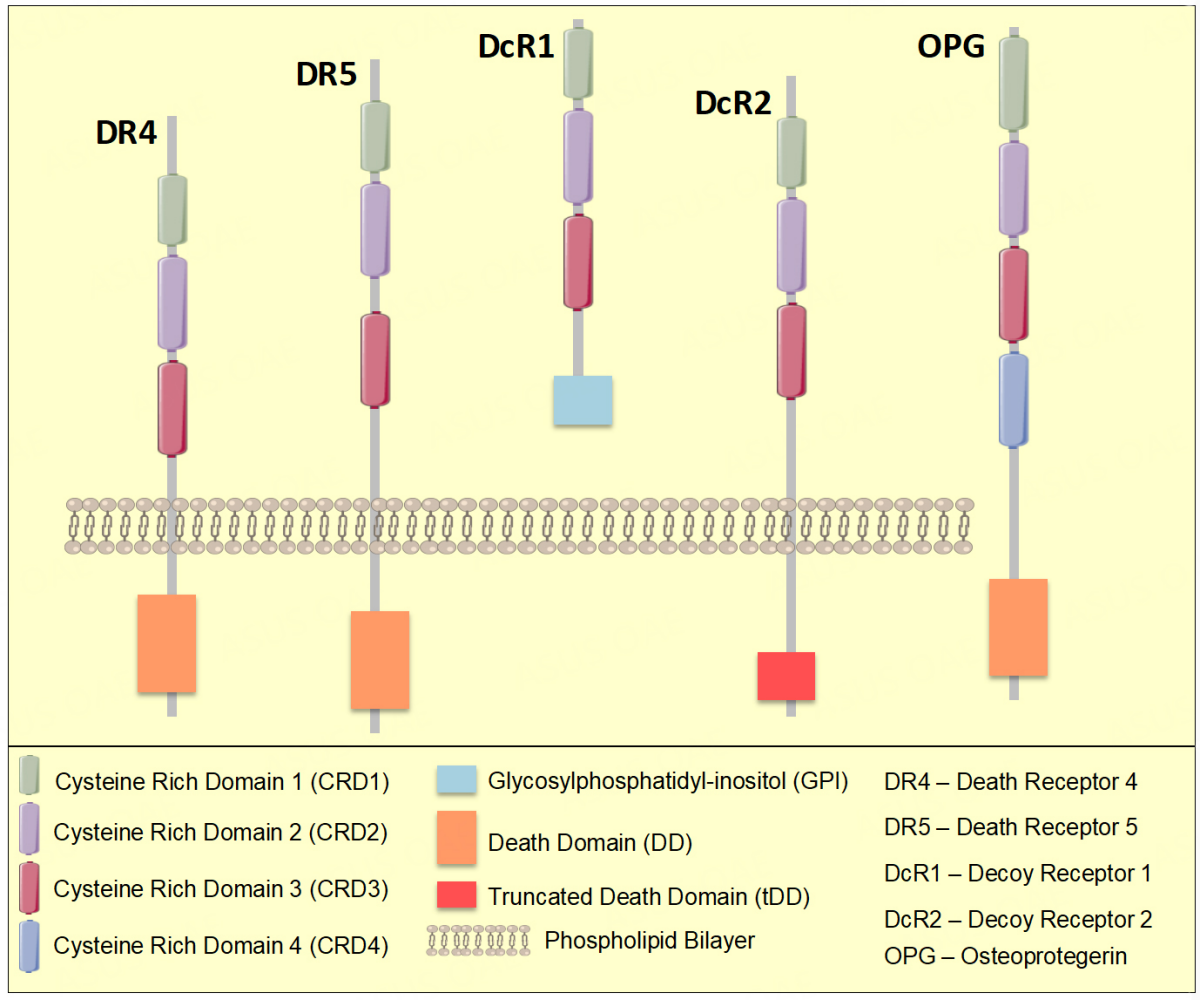
Structure of TRAIL receptors. TRAIL binds to death receptors DR4 (TRAIL-R1) and DR5 (TRAIL-R2) at the extracellular domains. The death domains (DD) on the cytoplasmic side lead to recruitment of other DD containing proteins that lead to apoptosis. TRAIL decoy receptors lack the cytoplasmic DD or have a truncated DD which fails to trigger apoptosis. OPG is a soluble receptor of TRAIL with low affinity.

TRAIL is a transmembrane protein belonging to the type II superfamily of ligands, coded by the gene TNFSF10 positioned at 3q26 on human chromosome number 3. In its monomeric form, TRAIL comprises 281 amino acids with a molecular mass of approximately 32.5 kDa. A loop formed by 12-16 amino acids near the N-terminus is critical for binding of TRAIL to its receptors and its cytotoxic activity^[11]^. TRAIL expression as a homotrimer is maintained by a zinc ion bound to cysteines necessary for its stability, solubility, and bio-activity^[12]^. TRAIL associates with DR4, DR5, DcR1, and DcR2, composed of a cysteine-rich domain (CRD), a transmembrane domain, and a death domain (DD), of which CRDs are extracellular, and DDs are intracellular [Figure 1]. The presence or absence of these domains characterizes TRAIL receptor structure and function. The type I transmembrane proteins (DR4 and DR5) are TRAIL agonists, whereas DcR1 and DcR2 are antagonists. DR4 and DR5 include an intracellular DD responsible for apoptosis induction. Unlike DR4 and DR5, DcR1 and DcR2 cannot trigger apoptosis due to the lack of a DD in the former and the presence of a non-functional truncated DD in the latter^[13]^. TRAIL also binds to osteoprotegerin (OPG), a receptor secreted as a soluble dimer. TRAIL binding to OPG occurs with low affinity. Other TNF family members bind with greater affinity to OPG, activating bone remodeling pathways^[14]^.

### TRAIL-induced apoptosis: extrinsic and intrinsic pathways

**Figure 2:**
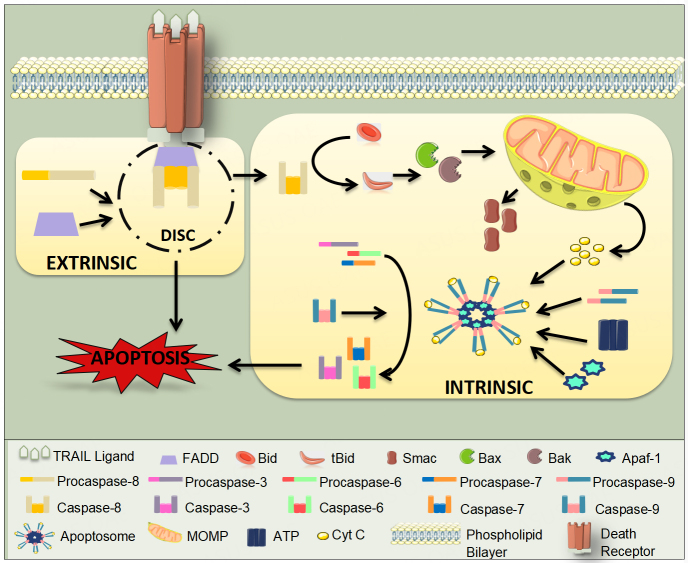
TRAIL-Induced Apoptosis: Cross talk between the Extrinsic and Intrinsic Pathways. TRAIL binding to death receptors leads to its trimerization on the cell surface and formation of DISC. Caspase-8 gets activated in the DISC and triggers the extrinsic pathway of apoptosis. Activated caspase-8 also leads to activation of the mitochondrial pathway or intrinsic pathway of apoptosis resulting in activation of caspase-9 and executioner caspases-3 and -7. Several checkpoints of apoptosis, like increase in anti-apoptotic proteins (IAPs), Bcl-xLetc are also represented at various stages of apoptosis. ATP: Adenosine triphosphate; DISC: Death Inducing Signaling Complex; FADD: Fas-associated death domain; MOMP: mitochondrial outer membrane permeabilization.

DR4 and DR5 are cell surface death receptors that include a cytoplasmic DD. In the extrinsic apoptosis pathway, TRAIL binds to its respective death receptors, resulting in the recruitment via homotypic interactions of the Fas-associated death domain (FADD), an adapter protein to their intracellular death domains. FADD then recruits initiator caspase-8 and caspase-10 to its death effector domain (DED) region, forming the multi-protein DISC. The activation of caspases-8 and -10 leads to cleavage and activation of effector caspase-3, -6, and -7, resulting in cell death [Figure 2]^[15-18]^.

DNA damage, loss of survival factors, and cell cycle checkpoint defects trigger the intrinsic apoptosis pathway; the extrinsic and the intrinsic pathways are interrelated. In the intrinsic pathway, there is a cleavage of BH3-interacting domain death agonist (Bid) to truncated Bid (tBid)^[19]^ in the presence of activated caspase-8 that brings about tBid translocation to the mitochondria and causes MOMP through activation of B-cell lymphoma 2 (Bcl-2) associated x-protein (Bax) and Bcl-2 homologous antagonist/killer (Bak). Mitochondria subsequently release cytochrome C^[20]^ and mitochondria-derived activator of caspase (Smac)^[21]^ into the cytosol, where cytochrome C interacts with adenosine triphosphate (ATP) and apoptotic peptidase-activating factor-1 (Apaf-1) to associate with initiator pro-caspase-9 into a signaling complex. This is an apoptosome where caspase-9 becomes activated and activates effector caspases-3, 6, and 7 to trigger apoptosis [Figure 2]^[8,10,22]^.

## TRAIL AND CANCER THERAPY

TRAIL is a potential candidate for targeted therapy in cancer due to its selective induction of apoptosis via death receptor-4 and -5 expressed on the surface of target cells. The two primary treatment methods are the administration of recombinant TRAIL protein (small peptide or full-length protein) or TRAIL-receptor (TRAIL-R) agonists^[23]^. However, the short half-life of TRAIL protein in serum and the inefficacious administration of the agonists *in vivo* have hampered its therapeutic use^[24]^. Moreover, many cancerous cells have been discovered to be TRAIL-resistant^[25]^. Several groups have tried to decipher the resistance mechanisms in various cancers and various ways to sensitize cells to TRAIL^[26-30]^. Several explanations have been offered for TRAIL resistance, some of which are discussed below.

### Mechanisms of cancer cell resistance to TRAIL

#### TRAIL receptor expression and status

**Figure 3:**
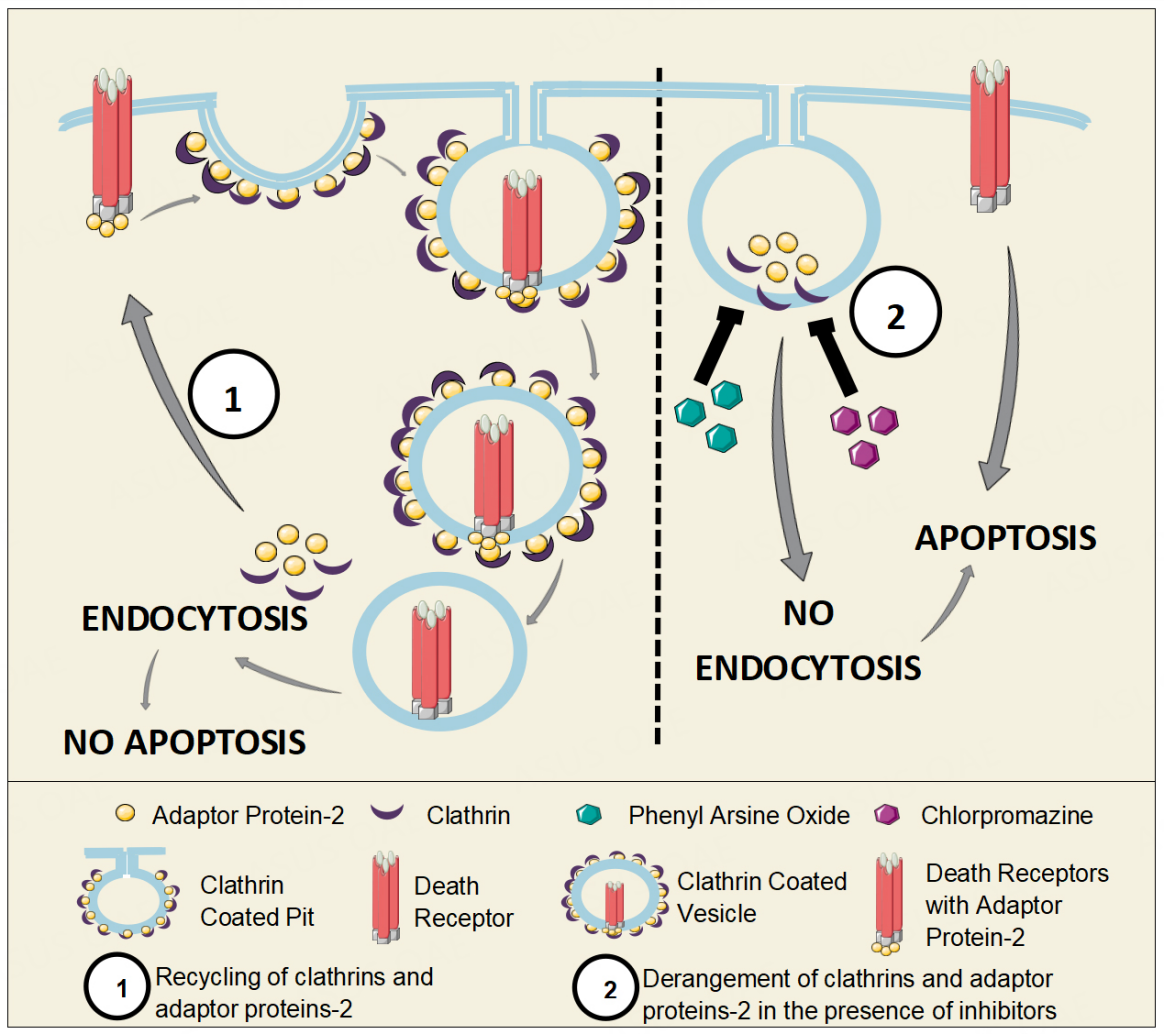
TRAIL Receptor endocytosis. Death receptors are constitutively recycled by clathrin mediated endocytosis. Lack of death receptors leads to TRAIL resistance. After endocytosis of the receptor, clathrin and adaptor proteins (AP2) are recycled to the cell membrane to continue with more endocytosis of the receptors. Pharmacological inhibitors like PAO or chlorpromazine prevent the arrangement of clathrin and AP2 at the vesicles, hence prevent endocytosis, resulting in persistent expression of the death receptors at the surface and increased apoptosis.

TRAIL induces apoptosis by binding to DR4 and DR5 death receptors. Loss of DR4 and DR5 expression because of constitutive endocytosis results in TRAIL resistance and is observed in some breast cancer cells. The comparison of six cell lines of human breast cancer (SKBR3, MDA-MB-468, MCF7, MDA-MB-231, T47D, and BT474) treated with recombinant human TRAIL (rhTRAIL) and antibodies against death receptor 4 (DR4) and death receptor 5 (DR5) for the apoptotic response showed the loss of expression of DR4 and DR5 in some cell lines that accounted to their antibody resistance. The derangement of clathrin-dependent endocytosis signaling elements (adapter protein 2 and clathrin) by pharmacologic inhibitors like phenyl arsine oxide, PAO (a general inhibitor of endocytosis), and chlorpromazine (an inhibitor of clathrin-mediated endocytosis) restored the death receptors’ expression on the cell surface, making TRAIL-resistant cells susceptible to TRAIL-induced apoptosis [Figure 3]^[31]^. In another study, DR5-B, a receptor-selective TRAIL variant, caused independent internalization of DR5; the comparison of the kinetics of TRAIL-mediated internalization and subsequent DR4 and DR5 recycling in sensitive (HCT116 and Jurkat) and resistant (HT-29 and A549) tumor cell lines of various origins revealed that TRAIL stimulated DR4 and DR5 receptor internalization in a dose-related manner. This expression of the receptor on the cell surface was restored after TRAIL internalization and elimination observed by the addition of cycloheximide and brefeldin A, which hindered the process, suggesting that death receptors undergo constitutive endocytosis^[32]^.

Apart from receptor endocytosis, mutations in death receptors^[33]^ and expression of decoy receptors lacking the functional intracellular DD may play a key role in apoptosis induced by TRAIL^[34]^. DcRs, especially decoy receptor 4, might compete for binding to TRAIL or may disrupt trimerization of the death receptors when co-expressed on the same cell^[35]^. Post-translational modifications of the receptors also influence the sensitivity of the cells to TRAIL. *O*-glycosylation of TRAIL-R2 was found to control cell sensitivity to TRAIL^[36]^, while *N*-glycosylation of TRAIL-R1 increased its apoptotic efficacy^[37]^.

#### FADD defects and caspase-8

Cancer cells require FADD (an adapter protein) and caspase-8 for apoptosis mediated by DR4 and DR5. FADD recruitment by TRAIL results in the activation of pro-caspase-8, DISC formation, and apoptosis [Figure 2]. Defective FADD proteins do not allow TRAIL to recruit FADD and inhibit initiator caspases activation, resulting in TRAIL resistance. Experiments on FADD-deficient^(-/-)^ mouse embryonic fibroblasts revealed that DR4 uses a FADD-independent apoptotic mechanism^[38]^. In another study, the transfection of mouse DR4/5, human DR4, or human DR5 into FADD-deficient mouse embryonic fibroblast cells conferred resistance to TRAIL-mediated cell death. However, they were vulnerable to TRAIL-mediated death when heterozygous FADD^(+/+)^ or FADD^(-/-)^ cells were reconstituted with a FADD retroviral construct^[39]^.

#### Overexpression of cFLIP

The non-functional pro-caspase homolog and an anti-apoptotic protein known as cellular Flice-like inhibitory protein (cFLIP) is crucial in controlling TRAIL-induced apoptosis^[40]^; cFLIP binds to FADD and prevents recruitment of caspase-8/-10 to DISC, thereby inhibiting their activation [Figure 2]^[41-43]^. In one study, DR5-positive and TRAIL-resistant IGR cells expressed enhanced TRAIL sensitivity with the downregulation of cFLIP. In TRAIL-sensitive and RPM-EP melanoma cells expressing DR5, TRAIL-mediated apoptosis inhibition was observed; due to the expression of cFLIP, TRAIL-R1 negative melanoma cells could not undergo apoptosis induced by TRAIL^[44]^. A study on epithelial-mesenchymal transition (EMT) showed that high expression of cFLIPs in the exogenous region caused resistance to apoptosis triggered by TRAIL, and deletion of cFLIPs was sufficient to overcome TRAIL resistance in carcinoma cell lines; when ML327 (an isoxazole-based small chemical) was induced into an immortalized mouse mammary epithelial cell line, there was a partial reversal of TGF-β-induced EMT^[45]^.

#### CARP-dependent degradation of active caspase-8

CARPs are caspase-8 and -10-associated ring proteins that belong to a class of apoptotic inhibitors that bind to and negatively control caspases. Active caspases are essential in cancer cell death. The overexpression of CARPs in cancerous cells leads to the degradation of active caspases, which in turn causes a rise in the levels of cFLIP and, subsequently, TRAIL resistance. In one study, parental DLD1 human colon cancer cells developed TRAIL resistance after siRNA-mediated suppression of caspase-8 expression. When caspase-8 protein expression was restored through stable transfection, the DLD1/TRAIL-R cell line regained its TRAIL sensitivity, suggesting that the DLD1/TRAIL-R cells’ TRAIL resistance may be due to low levels of caspase-8 protein expression. A 30%-50% increase in CARP-1 and -2 mRNA levels was observed in DLD1/TRAIL-R compared to a DLD1 cell line^[46]^. In another study, RNA interference-mediated silencing of CARPs resulted in H460 human lung cancer cell sensitization to death ligands due to activation of caspases; caspase-8 and -10 cleavage in reaction to the ligands were elevated in H460 cells after CARP-1 or -2 siRNA treatment and TNF-α/cycloheximide or TRAIL exposure^[47]^.

#### Loss of Bax/Bak function (Bax Mutations)

The Bcl-2 antagonist/killer (Bak), and Bcl-2-associated X-protein (Bax), members of the Bcl-2 family, are core regulators of the intrinsic apoptotic pathway. Bax and Bak oligomerize and form pores in the mitochondrial outer membrane through which cytochrome C is released [Figure 2]. Cytochrome C triggers the intrinsic pathway^[48-50]^. A study of TRAIL-resistant leukemia cells lacking Bax and Bak showed no release of apoptogenic proteins from mitochondria. When Bax was transduced into Bax/Bak deficient leukemic cells, they exhibited sensitivity to TRAIL^[51]^. Cells lacking functional Bax treated with the chemopreventive agent sulindac and other non-steroidal anti-inflammatory agents completely deactivated the apoptotic pathway in human colorectal cancer cells. Loss of Bax did not affect TRAIL-induced caspase-8 activation or Bid cleavage but inhibited the mitochondrial release of Smac/Diablo and cytochrome C^[52]^.

#### Bcl-2/Bcl-xL overexpression

B-cell lymphoma 2 (Bcl-2) and B-cell lymphoma-extra large (Bcl-xL) are the anti-apoptotic Bcl-2 family proteins that inhibit mitochondria-dependent cell death pathways by hindering the movement of Bax from the cytosol to mitochondria [Figure 2]. In response to TRAIL treatment, apoptosis was reduced by Bcl-2 overexpression, causing TRAIL resistance^[53-55]^. Combining TRAIL with the protein synthesis inhibitor cycloheximide improved the sensitivity of the vector control cells to the triggering of apoptosis in SH-EP neuroblastoma cells^[56]^. Overexpression of Bcl-2 and Bcl-xL resulted in the reduction of TRAIL-induced cleavage of caspase-8 and Bid, cleaved blockage of caspase-3, -7, and -9 in non-small-cell lung cancer (NSCLC) and the cleavage of caspase substrates like PARP in glioblastoma, neuroblastoma, and breast cancer cell lines^[57]^. Bcl-2 overexpression inhibited TRAIL-induced Bax activation, loss of MOMP, and cleavage of caspase-8 into p43/p41 and p18 active fragments, cleavage of Bid, and processing of the caspase-3 p20 fragment into the p17/p12 active fragments in SH-EP neuroblastoma cells^[58]^. Bcl-xL overexpression was found in T47D and SKBR3 breast cancer cell lines resistant to TRAIL. Silencing Bcl-xL increased TRAIL-induced activation of caspase-3/7 in the estrogen receptor-positive cell line T47D and the HER2-amplified cell line SKBR3^[59]^.

#### Overexpression of inhibitor of apoptosis proteins

Caspase activation is crucial for apoptosis. The family of anti-apoptotic proteins includes baculovirus repeat domain-containing proteins, also called inhibitor of apoptosis proteins (IAPs). These proteins consist of a (BIR) domain and a zinc ion binding site that coordinates protein-protein interaction and regulates the activity of caspases. IAPs cause TRAIL resistance by inhibiting the activation of caspases downstream [Figure 2], thus blocking cell death^[60-62]^. In one study, when rhTRAIL alone or combined with Smac mimetics GDC-0152 or birinapant was used to treat non-iodine-retaining follicular thyroid carcinoma (FTC) cell lines, FTC133 and TT2609-bib2 resulted in FTC cell lines sensitization to apoptosis induced by TRAIL through cIAP1/2 degradation^[60]^. In another study, TRAIL sensitivity was restored in resistant cells and primary leukemic blasts upon treatment with XIAP inhibitors. The impacts of numerous anti-leukemic drugs could be used to overcome TRAIL resistance in leukemic cells via XIAP downregulation^[63]^.

#### Reduction in smac/diablo release

A mitochondrial protein called the second mitochondria-derived activator of caspases (Smac) or direct inhibitor of apoptosis-binding protein with low pI (Diablo) binds to XIAP and eliminates the inhibitory effect of XIAP on caspase activation. Reduced release of Smac/Diablo causes a rise in the concentration of IAPs, inhibiting apoptosis^[64-66]^. SH122, a Smac-mimetic, significantly sensitized prostate cancer cell lines (DU145 and LNCaP) to TRAIL^[67]^. TRAIL-resistant lines associated with higher levels of XIAP released more Smac/Diablo than TRAIL-sensitive cells^[68]^.

#### Activation of various mitogen-activated protein kinases/nuclear factor-kappa B subunits

Mitogen-activated protein kinases (MAPK) and nuclear factor-kappa B (NFkB) show opposing activities concerning TRAIL-induced cell death. The association of truncated Bid (tBid) with mitochondria to release cytochrome C is inhibited by the MAPK pathway. NFkB activation results in elevated levels of the anti-apoptotic proteins Bcl-xL and XIAP, leading to TRAIL resistance. NFkB suppression decreased resistance to TRAIL in various cancers^[69,70]^. Lovastatin, an NFkB inactivator, sensitized human glioblastoma cell lines (A172 and U87) that were TRAIL-resistant. The sensitization to TRAIL is attributed to increased levels of DR5 and dysregulation of the MAPK pathway^[71]^; c-Rel, an NFkB subunit regulates TRAIL-induced apoptosis in pancreatic ductal adenocarcinoma cells and transfection with siRNA against c-Relinduced apoptosis in TRAIL-resistant cells^[72]^.

#### Akt pathway activation

The phosphatidylinositol 3-kinase-protein kinase B (PI3K-Akt) pathway is an intracellular signal transduction mechanism that stimulates cell growth and angiogenesis upon extracellular signals. Akt overexpression causes cancer cells to become highly resistant to TRAIL, while Akt knockdown causes resistant cancer cells to become more susceptible^[73]^. Inhibiting the Akt pathway with LY294002 (a PI3K inhibitor) or Akt knockdown sensitized TRAIL-resistant T47D breast cancer cells through cleavage of PARP^[74]^. Treatment of TRAIL-resistant MB-IT R and NALM-24 R (acute lymphoblastic leukemia cells) with LY294002 and TRAIL resulted in the activation of pro-caspase-8 and caspase-3^[75]^. Phosphatase and tensin homolog (PTEN), a tumor suppressor, negatively regulated the Akt pathway; PTEN-deficient cells were more resistant to TRAIL than PTEN(+/+) mouse prostate epithelial cells. Overexpressing a mutant PTEN made PTEN-positive cells resistant to TRAIL, suggesting that PTEN plays a part in TRAIL sensitivity. Overexpression of mutant PTEN showed increased resistance to TRAIL in T47D breast cancer cell lines resistant to TRAIL^[74]^. In SH-EP neuroblastoma cells, silencing the PI3K subunits p110 α and p110 β caused a selective downregulation in Akt phosphorylation. Combining TRAIL and PI103 (a PI3K inhibitor) led to an increase in broken caspases-8, -3, and -9, the conversion of Bid into tBid, and a decrease in the protein levels of Mcl-1, XIAP, survivin, cFLIPL, and cFLIPS^[58]^. El-Diery and colleagues described the role of PI3-Akt signaling in TRAIL- and radiation-induced gastrointestinal apoptosis. Activation PI3/Akt pathway protected gut cells from TRAIL-induced apoptosis but not from IR-induced apoptosis^[76]^. Akt activation reduced the sensitivity of epithelial ovarian cancer cells to TRAIL. PI3K or Akt inhibitors sensitized TRAIL-resistant SKOV3ip1 and COV2 cells. In TRAIL-resistant cells, low levels of Bid were observed due to overexpression of Akt^[77]^.

### Studies of TRAIL in cancer clinical trials

Because TRAIL triggers apoptosis specifically in cancer cells as opposed to tumor cells, several formulations of TRAIL protein and agonistic antibodies were tried in animal models and in clinical trials. Recombinant human TRAIL preferably promoted programmed cell death in cancerous cells over normal cells and exhibited minimal to no toxicity when injected systemically in animals^[78]^. This finding resulted in several treatment trials of biological agents targeting TRAIL receptors, including agonistic antibodies to DR4, DR5, and rhTRAIL^[79] ^[Table 1]. The clinical evaluation of TRAIL and its agonistic antibodies as anticancer treatments selectively killed cancerous cells^[94]^. Despite promising results in preclinical studies, TRAIL-based therapies carry several disadvantages.

**Table 1:** TRAIL-R agonists/recombinant TRAIL in clinical trials

**TRAIL-R agonists/ recombinant TRAIL**	**Mechanism**	**Cancer type**	**Clinical trials**	**References**
AMG 951 (Dulanermin) (recombinant human TRAIL)	DR4 and DR5 activation	NSCLC	Phase-II	NCT00508625^[80]^
AMG 951 (Dulanermin) (recombinant human TRAIL)	DR4 and DR5 activation	Metastatic colorectal carcinoma	Phase-I	NCT00873756^[81]^
AMG 951 (Dulanermin)(recombinant human TRAIL)	DR4 and DR5 activation	Advanced non-small-cell lung cancer	Phase-III	NCT03083743^[82]^
HGS-ETR1 (Mapatumumab) (TRAIL-R1 agonist)	Activation of caspase-8, -9, -3, Bid, cleavage of PARP	Multiple myeloma	Phase-II	NCT00315757^[83]^
HGS-ETR1 (Mapatumumab) (TRAIL-R1 agonist)	Activation of caspase-8, -9, -3, Bid, cleavage of PARP	Hepatocellular carcinoma	Phase-II	NCT01258608^[84]^
HGS-ETR1 (Mapatumumab) (TRAIL-R1 agonist)	Caspase-8 activation, -9, -3, Bid, cleavage of PARP	Advanced NSCLC	Phase-II	NCT00583830^[85]^
HGS-ETR1 (TRAIL-R1 agonist)	Caspase-8 activation, -9, -3, Bid, cleavage of PARP	Relapsed/refractory NHL	Phase-II	NCT00094848^[86]^
CS-1008 (Tigatuzumab) (TRAIL-R2 agonist)	Antibody-dependent cell cytotoxicity	Metastatic or unresectable NSCLC	Phase-II	NCT00991796^[87]^
CS-1008 (Tigatuzumab) (TRAIL-R2 agonist)	Antibody-dependent cell cytotoxicity	Pancreatic cancer	Phase-II	NCT00521404^[88]^
CS-1008 (Tigatuzumab) (TRAIL-R2 agonist)	Antibody-dependent cell cytotoxicity	Metastatic triple-negative breast cancer	Phase-II	NCT01307891^[89]^
CS-1008 (Tigatuzumab) (TRAIL-R2 agonist)	Antibody-dependent cell cytotoxicity	Advanced hepatocellular carcinoma, Hepatic cancer, Liver cancer, Liver neoplasms	Phase-II	NCT01033240^[90]^
AMG 655 (Conatumumab) (TRAIL-R2 agonist)	DR5 activation	Hodgkin’s lymphoma, Non-Hodgkin’s lymphoma, LowGrade lymphoma, Lymphoma, Diffuse large cell lymphoma, Mantle cell lymphoma	Phase-I	NCT00791011^[91]^
AMG 655 (Conatumumab) (TRAIL-R2 agonist)	DR5 activation	Metastatic pancreatic cancer	Phase-Ib/II	NCT00630552^[92]^
AMG 655 (Conatumumab) (TRAIL-R2 agonist)	DR5 activation	Metastatic colorectal cancer	Phase-Ib/II	NCT00625651^[93]^

A randomized phase II study showed that a dual DR4 and DR5 agonist called dulanermin did not enhance the response of NSCLC patients to standard chemotherapy (paclitaxel, carboplatin, and bevacizumab)^[95]^. In a phase II study, the combination of mapatumumab with carboplatin and paclitaxel in advanced NSCLC patients showed no clinical benefit in unselected patients. No improvements were seen in disease control rates, overall survival, or median progression-free survival upon adding mapatumumab^[96]^. Tigatuzumab, a TRAIL-R2 agonist and a humanized monoclonal antibody with sorafenib, was tested in patients with advanced hepatocellular carcinoma in a phase II randomized study of safety and tolerability. Tigatuzumab with sorafenib (compared with sorafenib alone) in individuals with advanced hepatocellular cancer did not fulfill its primary efficacy endpoint, i.e., time-to-progression, although tigatuzumab with sorafenib is well tolerated in hepatocellular carcinoma^[97]^. Tigatuzumab did not increase the effectiveness of carboplatin/paclitaxel in patients with advanced NSCLC^[98]^. In a phase II clinical trial, Tigatuzumab with nanoparticle-albumin-bound paclitaxel appeared to enhance apoptosis in patients with triple-negative breast cancer^[99]^. Conatumumab, a fully human monoclonal IgG1 antibody that activates DR5 in combination with 5-fluorouracil, leucovorin, oxaliplatin (mFOLFOX6) and bevacizumab (bev), was tested for safety, tolerability, and efficacy in previously-untreated metastatic colorectal cancer patients. In the primary treatment of metastatic colorectal cancer patients, conatumumab with mFOLFOX6/bev showed no better efficacy than the same chemotherapy with a placebo^[100]^.

The limited therapeutic potential is due to TRAIL resistance in several cancers^[101]^. The development of nanotechnology appears to be a promising strategy with several nano-based formulations of TRAIL and agonistic antibodies that help increase the circulating half-life and biodistribution of TRAIL in addition to its improved targeted delivery to tumor tissue.

## NANOPARTICLE-BASED DRUG AND GENE DELIVERY SYSTEMS

**Figure 4:**
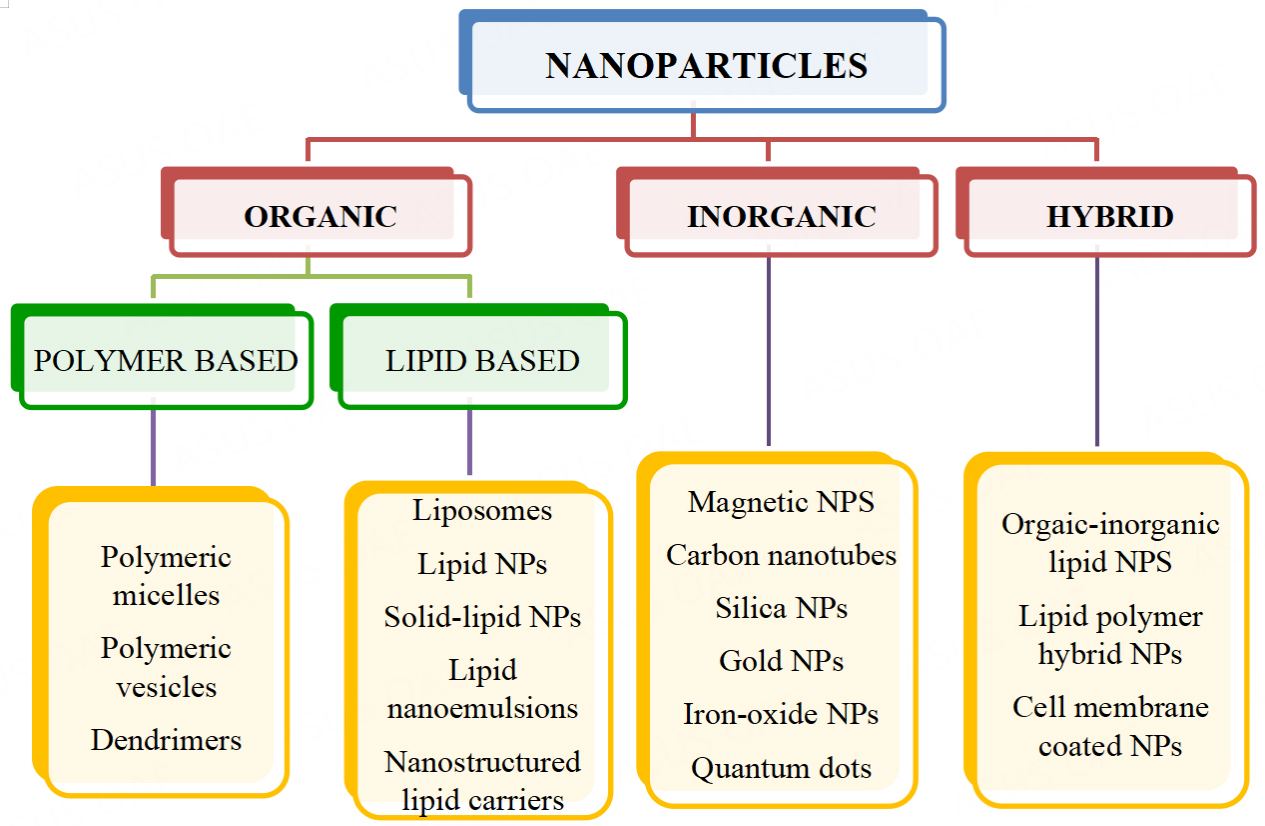
Types of Nanoparticles. Nanoparticles are classified based on the material used to prepare the various particles, organic, inorganic or hybrid. Different kinds of these formulations are represented here. NPs: Nanoparticles.

Nanoparticles (NPs), also known as ultrafine particles, are a wide class of materials with sizes ranging between 1 to 100 nanometers. At present, a wide variety of nanoparticles serve as drug and gene delivery systems due to improved permeability and retention effect, increased stability and biocompatibility, and precision targeting^[102]^. Nanoparticles can be classified into many types, one among them being organic and inorganic nanoparticles based on the addition of organic and inorganic elements, respectively [Figure 4]. There is another type of nanoparticles, the hybrid nanoparticles, the conjugates of organic and/or inorganic materials. These hybrid nanoparticles help to overcome the drawbacks of the conventional nanoparticulate delivery systems, such as low water solubility, non-specific targeting, and poor therapeutic outcomes^[103]^.In the below sections, we will give an overview of nanoparticle-based TRAIL therapy in different cancers. Nanoparticle formulations were tested in delivering recombinant TRAIL protein along with the TRAIL gene and the TRAIL receptor agonists in various models.

### Nanoparticle-mediated TRAIL drug delivery

The various nanoparticles formulated to deliver TRAIL protein are discussed in detail below and Table 2.

**Table 2:** Nanoparticle-based formulations of TRAIL protein for the treatment of various cancers

**Nanoparticle type**	**Cancer type**	**Mechanisms to enhance TRAIL sensitivity**	**Reference**
Single-walled carbon nanotubes	Colon adenocarcinoma, Squamous NSCLC, hepatocarcinoma, hepatoblastoma	Increased caspase-8 levels and enhanced DISC formation	[104]
Graphene nanosystem with doxorubicin	Lung & colon adenocarcinomas	Increased death receptor expression	[105]
Graphene quantum dots	Colon cancer	Increased pro-caspase-8 activation	[107]
Nanogold particles	Non-small cell lung cancer	Increased DR5 expression levels	[108]
Gold nanoparticles	Non-small cell lung cancer	Increased Drp1 recruitment to the mitochondria; Dysfunctioning of mitochondria	[109]
Silver-cysteine particles coated with polyethylene glycol	Colon cancer	Increased Bax and cleaved PARP; Decreased Bcl2 protein	[110]
Silver nanoparticles	Glioblastoma	Activation of death receptors; Increased caspase function	[111]
Double-edged lipid nanoparticles with doxorubicin	Hematological & epithelial carcinoma	Downregulation of FLIP and XIAP	[112]
LUV-TRAIL with doxorubicin	Breast cancer	Enhanced caspase-8 activation	[113]
LUV-TRAIL	Histiocytic lymphoma	Increased Bid, cleaved PARP, caspase-8, -3 & -10 levels; Enhanced DISC recruitment	[114]
LUV-type liposomes	Non-small cell lung cancer	Activation of caspase-8 & -3	[115]
LUV-TRAIL	Colon cancer	Increased caspase levels	[116]
Lipid nanoparticles	Hepatic fibrosis	Increased pro-apoptotic levels; Decreased uPA levels	[117]
Lumazine synthase protein cage nanoparticles	Epidermoid cancer	Intrinsic & extrinsic pathway activation	[118]
Ferumoxytol comprised of iron oxide nanoparticles	Colorectal cancer	Upregulation of DR5 levels; Overexpressed cleaved PARP	[120]
Magnetic ferric oxide nanoparticles	Glioma	Increased cleaved caspase-3 & cleaved PARP levels Increased pro-apoptotic potential	[121]
Maghemite nanoparticles	Breast & lung cancers	Increased pro-apoptotic potential Activation of caspase-3; Downregulation of Bcl-2 &BclxL; Upregulation of Bax& Bad; Increased death receptor levels	[122]
Iron oxide magnetic nanoparticles with actein	Non-small cell lung cancer	Activation of caspase-3; Downregulation of Bcl-2 &BclxL; Upregulation of Bax& Bad; Increased death receptor levels Increased pro-apoptotic potential	[123]
Iron oxide nanoclusters	Breast cancer	Increased pro-apoptotic potential Enhanced caspase-3 and -8 levels	[124]
SPION/TRAIL nanocomplex hydrogels	Glioblastoma	Enhanced caspase-3 and -8 levels Decreased survivin expression	[128]
NCL-240-loaded polymeric micelles	Ovarian cancer	Decreased survivin expression Increased caspase-3 & -7 levels; Decreased surviving & Bcl-2 levels	[129]
PEI-PLGA nanoparticle	Breast cancer	Increased caspase-3 & -7 levels; Decreased surviving & Bcl-2 levels Increased caspase-8 levels	[130]
Polymeric nanoparticles	Glioblastoma	Increased caspase-8 levels Increased caspase activity	[131]
Micellar nanoparticle with doxorubicin	Colorectal cancer	Increased caspase activity Increased caspase-3 &cleaved PARP levels	[132]
PCEC nanoparticles with curcumin	Colon cancer	Increased caspase-3 & cleaved PARP levels	[135]

#### Carbon-based nanoparticles

Carbon nanotubes, particularly single-wall carbon nanotubes (SWCNTs), are used in many physical and medicinal applications owing to their substantial flexibility, high mechanical resilience, and hydrophobicity. SWCNTs quickly diffuse in an aqueous medium. TRAIL-based SWCNT nanovectors showed greater efficiency than TRAIL alone in the induction of cancer cell death. Nanovectorization of TRAIL entails tagging TRAIL to SWCNTs to mimic membrane-bound TRAIL. His-tagged TRAIL was fused to SWCNT-pyrene-butyric acid N-hydroxysuccimide ester (PSE)-polyethylene glycol (PEG) to produce functional nanoparticles. The pro-apoptotic potential of these SWCNT-PSE-PEG-TRAILs (also called NPTs) is increased by almost 20-fold in various human tumor cell lines (HCT116- colon adenocarcinoma cells, H1703-squamous NSCLC cells, HepG2-hepatocarcinoma cells, and HUH-hepatoblastoma cells) due to increased levels of caspase-8 and enhanced DISC formation^[104]^.

Another form of carbon-based nanoparticles is made from graphene, an allotrope of carbon. A graphene-based co-delivery nanosystem composed of graphene oxide, a PEG linker, and a furin-cleaved peptide encapsulated with TRAIL and doxorubicin (Dox) resulted in the efficient release of TRAIL and Dox to their sites of action by digesting the peptide linker by furin with the increased expression of death receptors in lung and colon adenocarcinoma cells^[105]^.

Docking studies of TRAIL nanovectorization were performed using graphene nanoflakes as a potential cargo for TRAIL. These studies showed that when adsorbed on graphene, TRAIL self-assembling and TRAIL affinities enhanced efficacy towards the targeted cancer cell. TRAIL bound to DR4 and DR5 more effectively when transported by graphene nanoflakes^[106]^. The synthesis of a novel nanohybrid system, sTRAIL (TRAIL fused with crystalline bacterial cell surface layer (S layer) protein), combined with graphene quantum dots or GQDs elevated the functional stability of TRAIL by improving pro-caspase-8 activation and mitochondria-dependent cell death in doxorubicin-pretreated human colon cancer cells (HT-29) when compared to S-TRAIL alone. The GQDs appeared to offer a surface for the S-TRAIL to self-assemble, facilitating TRAIL monomer oligomerization and enhancing apoptosis^[107]^.

#### Gold nanoparticles

Nanogold-TRAIL complexes are nanogold coated with TRAIL protein. In the NSCLC microenvironment, M2 macrophages exhibit anti-inflammatory and pro-tumorigenic effects, while M1 macrophages exhibit antitumorigenic effects. Elimination of the M2 macrophages aids tumor destruction; hence, these M2 macrophages serve as potential targets. *In vitro* targeting M2 macrophages (derived from THP-1 monocytes) with nanogold-TRAIL particles led to 30-fold higher cytotoxicity. This enhanced cytotoxicity is attributed to increased surface-level DR5 expression and changes in the O-glycosylation pattern of the death receptor^[108]^. AuNPs (other nanoparticles made of gold) enhanced TRAIL sensitivity through dynamin-related protein 1 (Drp1)-mediated apoptotic and autophagic mitochondrial fission in NSCLC cells. AuNPs with TRAIL exhibited greater potency to promote apoptosis in NSCLC cells than TRAIL alone due to a rise in mitochondrial recruitment of Drp1 that led to dysfunctioning of mitochondria and induction of autophagy^[109]^.

#### Silver nanoparticles

Experiments with silver-cysteine nanoparticles and TRAIL-conjugated silver nanoparticles coated with PEG (AgCTP NPs) showed that AgCTPNPs exhibited apoptotic effects in HT-29 colon cancer cell line in vitro with substantial differences in the expression levels of Bax, Bcl-2, and cleaved PARP protein^[110]^. TRAIL-conjugated AgNPs (TRAIL-AgNPs) induced death receptor activation in T98 G TRAIL-Resistant (TR) glioblastoma cells. The combination of TRAIL to silver nanoparticles (AgNPs) increased the functioning of caspases in TR glioblastoma cells, but TRAIL and AgNPs alone did not. Moreover, TRAIL-AgNPs-treated TR cells exhibited lower CHK1 expression than TRAIL-treated cells^[111]^.

#### Lipid-based nanoparticles

Membrane-bound TRAIL in lipid nanoparticles, also known as large unilamellar vesicles (LUV)-TRAIL showed increased cytotoxic activity *in vitro* compared to soluble recombinant TRAIL in human sarcoma cells. LUV-TRAIL and anticancer drugs showed increased cytotoxicity against human sarcoma cells. LUV-TRAIL combined with flavopiridol (FVP) reduced human sarcoma cells’ long-term clonogenic survival and triggered apoptosis by downregulating FLIP and XIAP and activating caspases, compared to soluble TRAIL (sTRAIL) alone^[112]^. LUV-TRAIL loaded with doxorubicin (Dox) in the liposomal lumen, known as LUVDOX-TRAIL, improved cytotoxic potential by enhanced caspase-8 activation in the tumor xenograft model of breast cancer cells^[113]^.

LUV with tethered recombinant TRAIL (rTRAIL) was prepared and tested against U937 histiocytic lymphoma cells. LUV-TRAIL-induced cell death on U937 cells by causing cleavage of caspases faster than sTRAIL. Levels of Bid, cleaved PARP-1, caspase-8, -3, and -10 increased and enhanced recruitment of DISC upon treatment with LUV-TRAIL^[114]^.

LUV-type liposomes coated with human sTRAIL were also used to treat NSCLC. Treatment of A549 cells with LUV-TRAIL activated caspases-8 and -3, compared with sTRAIL. LUV-TRAIL combined with antitumor medications such as CDK inhibitors, FVP, and SNS-032 further sensitized A549 NSCLC cells to LUV-TRAIL-induced apoptosis^[115]^. In HCT-116 and HT29 colon cancer cells, DR5 was more effectively activated by TRAIL-coated lipid nanoparticles (LUV-TRAIL) than by sTRAIL. When cells were exposed to various types of TRAIL, the pan-caspase inhibitor Z-VAD-FMK completely blocked cell death, suggesting that LUV-TRAIL-induced cell death is a caspase-dependent mechanism^[116]^. Lipid nanoparticles embedded with recombinant human TRAIL gave rise to significant apoptosis in hepatic fibrosis. Levels of the pro‐apoptotic proteins were highly expressed, and the anti‐apoptotic protein (uPA) levels were significantly decreased by a self‐made drug carrier pPB‐SSL with rhTRAIL protein^[117]^.

#### Lumazine nanoparticles

A multiple ligand-displaying nanoplatform, lumazine synthase protein cage nanoparticle isolated from Aquifexaeolicus (AaLS) with TRAIL and EGFR binding affibody (EGFRAfb) via a SpyTag/SpyCatcher protein-ligation system (to form AaLS/TRAIL/EGFRAfb) exhibited TRAIL-mediated apoptosis in TRAIL-resistant and EGFR-overexpressing A431 epidermoid cancer cells *in vitro* through synergistic activation of the extrinsic and intrinsic apoptotic pathways^[118]^.

#### Albumin nanoparticles

Albumin-bound NP technology was modified slightly to create TRAIL/Dox human serum albumin (HSA)-NPs, loaded with Dox and TRAIL nanoparticles. Using HCT116 colon cancer cells, the synergistic cytotoxicity and apoptotic activity of TRAIL/Dox HSA-NPs were assessed. Results from tumor tissues demonstrated that TRAIL/Dox HSA-NPs significantly increased apoptosis, while Dox HSA-NPs did so at a slightly lower level^[119]^.

#### Magnetic/Iron oxide nanoparticles

Reactive oxygen species play critical roles in TRAIL-induced apoptosis. Iron oxide nanoparticles (iron oxide NPs) with ferumoxytol as its primary component spontaneously unite to form nanocomplexes with TRAIL/Apo-2L (NanoTRAIL). An investigation of the cell viability of HCT-116 (TRAIL-sensitive), SW-480 (TRAIL-intermediately resistant), and HT-29 (TRAIL-resistant) after treatments with saline, Apo-2L, iron oxide NPs, and NanoTRAIL resulted in a more modest TRAIL/Apo-2L response in HT29 than with other cell lines; there was a more significant anti-colorectal cancer tumor effect than with TRAIL/Apo-2L treatment alone in HT-29 cells with the upregulation of DR5 expression increased TRAIL/Apo-2L sensitivity and significant apoptosis. TRAIL sensitivity in the TRAIL/Apo-2L-resistant HT29 cells was due to activating JNK and increasing the expression of cleaved PARP^[120]^.

Treating U251 cell-derived glioma xenografts with TRAIL conjugated to magnetic ferric oxide nanoparticles resulted in increased apoptosis, decreased tumor volume, and more prolonged survival. Conjugation of TRAIL to NP showed higher apoptosis levels than free recombinant TRAIL by increasing caspase-3, -8, and cleaved PARP levels^[121]^. Maghemite nanoparticles coated with TRAIL protein (CO-TRAIL@NPs-NH and NH-TRAIL@NPs-CO) enhanced apoptosis in human breast (MDA-MB-231) and lung (H1703) carcinoma cell lines compared to free TRAIL by increasing the pro-apoptotic potential^[122]^.

Iron oxide (Fe_3_O_4_) magnetic nanoparticles combined with actein, a triterpene glycoside isolated from the rhizomes of Cimicifuga foetida, contributed to apoptosis in NSCLC. The induction of apoptosis in NSCLC cells resulted in the stimulation of the caspase-3 signaling pathway, which was identified by a decrease in levels of the anti-apoptotic proteins Bcl2 and Bcl-xL, increase in the pro-apoptotic signals Bax and Bad, and elevated levels of death receptors^[123]^. Interestingly, iron oxide nanoclusters (NCs) exhibited a synergistic effect with the TRAIL receptor. The engraftment of TRAIL onto NCs increased pro-apoptotic potential via nanoparticle-mediated magnetic hyperthermia (MHT) or photothermia in MDA-MB-231 wild-type and TRAIL receptor-deficient cells^[124]^.

When compared to free recombinant TRAIL, magnetic ferric oxide nanoparticles enhanced apoptosis activity against several human glioma cells. There were NP-TRAIL molecules at the tumor site and a notable increase in glioma cell apoptosis in U251-derived glioma xenografts, which were examined for their effects on programmed cell death, tumor volume, survival, rhodamine-tagged NPs, and xenografts^[125]^.

#### Polymer-based nanoparticles

Elastin-like polypeptides (ELPs) are potential thermosensitive biopolymers and polymeric drug delivery systems. The ability to induce apoptosis was three times greater when RGD-TRAIL was expressed coupled with ELPs than when RGD-TRAIL was expressed alone. When a single dose of the RGD-TRAIL-ELP nanoparticle was administered intraperitoneally, the COLO-205 tumor xenograft model showed nearly total tumor shrinkage. Protein electrophoresis revealed a 3.4-fold increase in the RGD-TRAIL-ELP trimer content than RGD-TRAIL^[126]^. P(RGD) proteinoids (where RGD is a tripeptide) comprising arginine, glycine, and aspartic acid, and proteinoid nanocapsules (NCs) were synthesized for targeted tumor therapy. TRAIL-P(RGD) nanocapsules induced cytotoxicity in CAOV-3 ovarian cancer cells more so than hollow P(RGD) nanocapsules which are atoxic in CAOV-3 cells. Dox-encapsulated P(RGD) nanocapsules also showed significant cytotoxicity in CAOV-3 cells^[127]^.

SPION/TRAIL nanocomplex hydrogels, an MHT-mediated TRAIL release system with positively-charged TRAIL and hydrophobic superparamagnetic iron oxide nanoparticles (SPIONs) complexed with negatively-charged poly(organophosphazene) polymers via ionic and hydrophobic interactions, is used for combination therapy for multiple mild MHT and simultaneously MHT-induced TRAIL release. Hyperthermia restored the sensitivity of intrinsic TRAIL-resistant human glioblastoma (U87 MG) cancer cells and enhanced TRAIL-induced apoptosis by activating caspases-3 and -8^[128]^.

A multifunctional nanoparticle system with a combination of a pro-apoptotic drug (NCL-240), TRAIL, and anti-survivin siRNA was used to test anticancer effects in various cancer cells. The NCL-240-loaded polymeric micelles (mNCL-240) and the combination of NCL-240-loaded, TRAIL-conjugated polymeric micelles (mNCL-240-loaded/TRAIL) showed significantly more cytotoxicity than single-drug formulations in all tested cell lines. Treating A2780 and U-87 MG cells with transferrin polymer micelles (Tf PM) improved the cytotoxic activity of NCL240 formulations, especially at higher doses. It was observed that mSurvivin (anti-survivin siRNA-S-S-PE mixed micelles) significantly downregulated survivin expression in ovarian cancer cells, A2780, compared to free anti-survivin siRNA and scrambled siRNA^[129]^.

Hyaluronic acid (HA)-decorated polyethylenimine-poly(D,L-lactide-co-glycolide) (PEI-PLGA) nanoparticle (NP) (also known as HA/PPNP) with gambogic acid (GA) and TRAIL plasmid (pTRAIL) showed synergistic effects against breast cancer cells. Increased caspases-3 and -7 were observed in MDA-MB-231 cells treated with GA-HA/PPNPs, pTRAIL-HA/PPNPs, and GA/pTRAIL-HA/PPNPs by 2.39-, 1.18-, and 4.55-fold, respectively, after treatment of cells for 24 h compared to HA/PPNPs alone. Increased caspase-8 levels were also observed with pTRAIL-HA/PPNPs and GA/pTRAIL-HA/PPNPs by 1.47- and 1.55-fold, respectively, compared to untreated controls. Cells treated with GA/pTRAIL-HA/PPNPs showed the lowest expression levels of survivin and Bcl-2 anti-apoptotic proteins^[130]^. The polymeric nanoparticle-engineered human adipose-derived stem cells (hADSCs) overexpressing TRAIL served as drug-delivery vehicles for targeting and eliminating glioblastoma multiforme (GBM) cells *in vivo*. The co-culturing of plasmid human TRAIL-laden nanoparticles (NPs/pTRAIL)-engineered hADSCs with patient-derived malignant glioma xenograft cells (D-270 MG) resulted in significant apoptosis and death in glioma cells (compared to controls) through an increase in levels of caspase-8 and the caspase cascade^[131]^. The delivery of micellar nanoparticles self-assembled from a biodegradable cationic copolymer P(MDS-co-CES) with TRAIL and Dox showed synergistic cytotoxic effects in TRAIL-resistant SW480 colorectal cancer cells. In cells treated with nanoparticles, in the presence of ZVAD-FMK, a pan-caspase inhibitor gave rise to TRAIL sensitivity, suggesting that apoptosis depends on caspase activity^[132]^.

N-{[(2-hydroxy-5-nitrophenyl)amino] carbonothioyl}-3, 5-dimethylbenzamide (DM-PIT-1)-loaded polyethylene glycol and phosphatidylethanolamine (PEG-PE) micelles modified with TRAIL were used against TRAIL-resistant U87MG cells. In contrast to PEG-PE micelles loaded with DM-PIT-1, drug-free PEG-PE micelles modified with TRAIL are not cytotoxic for U87MG cells. The synergistic effect and the significantly increased cell death were caused by the extra attachment of TRAIL to drug-loaded micelles. Compared to groups treated with typical DM-PIT-1-loaded micelles, all groups treated with TRAIL-modified DM-PIT-1-loaded PEG-PE micelles significantly reduced viability in U87MG cells^[133]^. PEGylated heparin, poly-L-lysine nanoparticles with TRAIL called TRAIL-PEG-NPs, had apparent apoptotic effects when administered *in vitro* and *in vivo*. TRAIL-PEG-NPs induced time-dependent apoptotic cell death and efficiently suppressed mean tumor growth with mean tumor growth inhibition^[134]^. The hydrophobic drug curcumin (Cur) encapsulated into the inner core of biodegradable poly (e-caprolactone)-poly (ethylene glycol)-poly (e-caprolactone) nanoparticles with TRAIL protein (TRAIL-Cur-NPs) was evaluated for their efficacy to treat HCT116 human colon carcinoma cells. Compared to the free TRAIL + Cur group, protein expression of cleaved caspase-3 and cleaved PARP was slightly higher in the TRAIL-Cur-NPs group. The overexpression of DR4 and DR5 synergistically increased the anticancer efficacy of TRAIL and Cur^[135]^. TRAIL-modified and cabazitaxel (CTX)-loaded polymer micelles (TRAIL-M-CTX) were synthesized to test the anticancer efficacy against TRAIL-resistant MCF7 breast cancer cells. Synergistic effects of apoptosis were observed in MCF7 cells treated with TRAIL-loaded nanoparticles than in CTX and TRAIL alone treated cells^[136]^.

### Nanoparticle-mediated TRAIL gene delivery

Several studies described the use of TRAIL protein-based nanoparticles. These trials have been successful but are limited by the relatively short half-life of the ligand, targeted delivery to the tumor cells, and proper presentability to the death receptors on the membrane. Hence, novel approaches have been attempted, one of which is delivering the TRAIL gene to the tumor cells instead of the protein^[137]^. This section summarizes studies of TRAIL gene-based nanoparticles [Table 3].

**Table 3:** Nanoparticle-based formulations of TRAIL gene for the treatment of various cancers

**Nanoparticle type**	**Cancer type**	**Mechanisms to enhance TRAIL sensitivity**	**Reference**
PEI-coated superparamagentic iron oxide nanoparticles with cisplatin	Ovarian cancer	Cytochrome-C release and caspase-9 cleavage	[139]
Chitosan magnetic nanoparticles	Melanoma	Activation of caspase-3	[140]
PEI-capped gold nanoparticles	Hepatoma	Increased stTRAIL’s mRNA and protein levels; Increased caspase-8 and -3 levels	[142]
Zein nanoparticles	Liver cancer	Increased p53 expression; Decreased MMP-2 expression	[143]
NIR light absorbing conjugated polymer nanoparticles	Breast cancer	Increased caspase-8 cleavage	[144]
Poly(beta-amino ester) nanoparticles	Liver cancer	Increased DR5 expression	[145]
Triazine modified PAMAM dendrimer	Osteosarcoma	Increased caspase-3, -7, cleaved PARP levels	[149]
RRPHC polymeric nanoparticles	Melanoma	Increased caspase-9 & -3 levels	[150]
Poly(beta-amino ester) nanoparticles	Lung cancer	Increased DR4 & DR5 expression levels	[151]
RRPHC nanoparticles	Colorectal cancer	Increased cleaved caspase-9 & -3 levels Increased levels of cleaved caspase-3 & -9 levels	[152]
PEI-R8 heparin nanogel	Colon cancer	Increased levels of cleaved caspase-3 & -9 levels	[153]
γ-PGA/Moβ-CD-SSPEI pDNA nanocomplexes	Colon & cervical cancers	Increased DR5 & cleaved PARP levels	[154]
PEI-CD/Ad-Dox/pDNA supramolecular nanoparticles	Ovarian cancer	Increased apoptotic protein levels	[155]
LCPP nanoparticles	Hepatocellular carcinoma	Increased DR5 expression phosphorylation	[157]
Lipid-Protamine-DNA nanoparticles		Decreased mTOR phosphorylation; Increased AMPK-alpha	[158]
Cationic albumin-pegylated nanoparticles	Glioma	Increased cleaved p17 fragment; Increased caspase-3 levels	[160]

#### Magnetic/Iron oxide nanoparticles

The TRAIL gene combined with polyethyleneimine-coated magnetic iron oxide nanoparticles (also known as polyMAG-1000) triggered significant apoptosis in MCF-7 breast cancer cells^[138]^.

The transfection of the TRAIL gene with polyethyleneamine-coated polyMAG-1000 combined with anticancer drug cisplatin/cis-diamminedichloroplatinum(II) (CDDP) triggered apoptosis in A2780/DDP ovarian cancer cells. Compared to TRAIL gene transfection or CDDP therapy alone, more apoptosis was seen in the presence of the medication. Cytochrome C release and the caspase-9 cleavage pathway were linked to the triggering of apoptosis in A2780/DDP cells^[139]^.

GBM is among the most aggressive types of brain cancer. The successful delivery of TRAIL-encoded plasmid DNA into human T98G GBM cells with chitosan-polyethylene glycol-polyethyleneimine copolymer and CTX-coated iron oxide nanoparticles successfully induced a three-fold increase in apoptosis compared to control cells^[24]^.

A nanosystem for magnetofection was used to create magnetic nanoparticles carrying the TRAIL gene and chitosan. In a melanoma pulmonary metastatic mouse model, the mouse melanoma cell line B16F10 was injected, and the mice were monitored for metastatic tumor formation in the lungs. These animals were then given a systemic injection of the abovementioned nanoparticles through the tail vein. Nanoparticles reaching the lungs were activated to express the TRAIL gene by an external magnetic field applied at the rib cage. There was a significant increase in tumor cell death and suppression of the development of metastatic melanoma^[140]^. In another study, the authors constructed a Fe_3_O_4_-PEI-plasmid complex (FPP) in which positively-charged PEI altered iron oxide nanoparticles to enable them to carry the negatively-charged plasmid pACTERT expressing TRAIL. *In vitro* and *in vivo* apoptosis of SACC-83 adenocystic carcinoma cells was successfully induced by FPP-mediated TRAIL gene transfer.

A substantial increase in apoptosis was observed in transfected SACC-83 cells. An analysis of anticancer properties of the magnetic complex-TRAIL in mice showed significantly smaller tumor size after transfection than the control groups^[141]^.

#### Gold nanoparticles

The combination of the secretable trimeric Tumor Necrosis Factor-Related Apoptosis-Inducing Ligand (stTRAIL) gene and PEI-capped gold nanoparticles (AuNPs) increased the inhibition of cell proliferation and promoted apoptosis in heat-shocked hepatoma cells; there was increases in the levels of stTRAIL mRNA and protein and caspases-8 and -3^[142]^.

#### Zein nanoparticles

Zein is a protein derived from maize that is prolamine-rich and is used as a nanocarrier because of its distinct structure, physicochemical characteristics, and self-assembly process. The growth rate and cell viability of HepG2 liver tumor cells treated with TRAIL-loaded zein nanoparticles (ZNPs) were lower than untreated cells in a concentration-dependent manner. Treatment with TRAIL genes packaged into ZNPs resulted in less MMP-2 expression in liver homogenates than untreated ones^[143]^.

#### Nano-polymers

Conjugated polymer nanoparticles (CPNs) that absorb near-infrared light were developed to remotely control and induce TRAIL-mediated apoptotic signaling to boost apoptosis in TRAIL-resistant cancer cells^[144]^. In response to heat shock, the promoter of a heat shock protein initiated transcription of the TRAIL gene; as a result, breast cancer cells expressed the TRAIL protein, activating the TRAIL-mediated death signaling pathway. The CPNs simultaneously produce W-7 (a calmodulin antagonist) which promotes caspase-8 cleavage and increases cancer cell death. *In vitro* and *in vivo* tests revealed that CPNs/W-7/p TRAIL has a considerable synergistic therapeutic effect on breast cancer and is barely toxic when exposed to near-infrared light. In HepG2 cancer cells, poly(beta-amino ester) nanoparticles (PBAE NPs), engineered with a cDNA sequence coding a secretable TRAIL protein or sTRAIL, initiated apoptosis by increased DR5 expression on the cell surface and a 40-fold increase in cell death, reprogramming liver cancer cells to produce TRAIL protein^[145]^. The combination of positively-charged polymer, PEI-modified Fe_3_O_4_ magnetic nanoparticles, and negatively-charged pACTERT-EGFP through electrostatic interaction resulted in a new magnetic nanovector (pACTERT-TRAIL) with antitumor properties in cell lines and xenograft models of oral squamous cell carcinoma^[146]^.

Polyamidoamine (PAMAM) dendrimers are distinctive nanostructures and are effective non-viral carriers because of their highly branched, three-dimensional structure with high loading capacity and lack of toxicity. Modifying G4 and G5 PAMAM dendrimers with alkyl carboxylate-PEG and cholesterol triggered apoptosis in colon cancer cells *in vitro* and *in vivo*. *In vivo*, there was a significantly higher therapeutic index of the plasmid TRAIL in modified PAMAM dendrimer (pTRAIL) than in unmodified PAMAM. Cells treated with the PAMAM G4 derivative coupled with alkyl-PEG and cholesterol (F5-G4) and TRAIL (F5-G4-TRAIL) and PAMAM G5 derivative with alkyl-PEG and cholesterol (F5-G5) and TRAIL (F5-G5-TRAIL) demonstrated 20.1% and 24.14%, of cell death, respectively^[147]^.

The combination of D-α-tocopheryl polyethylene glycol 1000 succinate-b-poly (ε-caprolactone-ran-glycolide), also known as TPGS-b-(PCL-ran-PGA) (a biodegradable diblock copolymer), PEI, and TRAIL showed anticancer effects *in vitro* and *in vivo* in HeLa cells. SCID mice carrying HeLa tumor xenografts showed increased cytotoxicity when treated with nanoparticles loaded with TRAIL^[148]^. Triazine, a molecule with DNA binding capacity, modified on a PAMAM dendrimer tagged with green fluorescence protein and TRAIL reporter gene (G5-DAT66/pTRAIL complex), was constructed and treated against an osteosarcoma cell line (MG-63). Higher levels of apoptosis were observed in cells treated with dendrimer and TRAIL complexes than in cells treated with dendrimer or TRAIL alone, with increased levels of caspase-3, -7, and cleaved PARP^[149]^. The design of core-shell ternary systems consisting of fluorinated polymers (PFs) binding with a plasmid (pDNA) and a negatively-charged multifunctional RRPH (arginyl-glycyl-aspartic acid (RGD)-cell-penetrating peptide (R8)-PEG-sodium hyaluronate/(RGD-R8-PEG-HA) shell, also known as RRPH/PF/pDNA (RRPHC) ternary complex or HA (sodium hyaluronate) shell, also known as HA/PF/pDNA (HAC) ternary complex for the delivery of pUNO1-mTRAIL (mouse TRAIL) plasmid was successful in causing apoptosis in B16F10 mouse melanoma cell line. Transfection with the complexes containing mTRAIL significantly inhibited the growth of the B16F10 cells and induced elevated apoptosis effect with increased expression levels of pro-apoptotic proteins (cleaved caspase-9 and cleaved caspase-3). The RRPHC/mTRAIL complexes induced a comparable apoptosis effect higher than that of HAC/mTRAIL ternary complexes^[150]^.

Several human cancer cell cultures can be transfected *in vitro* by DNA-containing polymeric nanoparticles based on PBAEs, leading to cell death. Recombinant human TRAIL (rhTRAIL) was not as successful as the PBAE/TRAIL-DNA therapy in killing H446 lung cancer cells, which also demonstrated high levels of DR4 protein expression, high levels of DR5, and low levels of DcR1 and DcR2 expression^[151]^. According to a study on the anticancer activity of the spontaneous nucleus-targeting core PF33/hTRAIL, HAC/hTRAIL, and RRPHC/hTRAIL complexes on HCT116 colorectal cancer cells, the TRAIL protein was significantly more highly expressed in the groups treated with PF33/hTRAIL and RRPHC/hTRAIL than in the groups treated with HAC/hTRAIL. The expression of cleaved caspase-9 and -3 proteins showed similar results, suggesting that the hTRAIL is responsible for apoptotic action^[152]^. A novel PEI-RRRRRRRR (R8)-heparin (HPR) nanogel with a plasmid containing human TRAIL gene (HPR/phTRAIL) showed higher levels of cleaved caspase-3 and -9 in HCT-116 colon cancer cells. HPR nanogel delivered phTRAIL into HCT-116 cells to express hTRAIL protein and significantly induced apoptosis^[153]^.

#### Carbon-based nanoparticles

In HCT8/ADR and HeLa cells, the cytotoxic activity of pDNA nanocomplexes containing PGA/Mo-CD-SSPEI and redox-sensitive bioreducible PEI with plasmid TRAIL was assessed in the presence and absence of monensin. By increasing DR5 and cleaved PARP levels, the pTRAIL-induced apoptotic rate in HCT8/ADR and HeLa cells was greater than that of the control groups. These findings suggest that the RGD-PGA/Mo-CD-SSPEIpTRAIL and PGA/Mo-CD-SSPEIpTRAIL groups had significant levels of TRAIL protein expression in the tumors. The dual-targeting RGD-PGA surface coating provided the best cancer cell treatment efficacy^[154]^.

Dox and TRAIL therapeutic gene-loaded PEI-CD/Ad-Dox/pDNA supramolecular nanoparticles (SNPs) were assayed for treating ovarian cancers. The PEI-CD/Ad-Dox SNP included in the pTRAIL prevented ovarian tumor growth *in vivo* and significantly increased the survival time of tumor-bearing mice. Free medication and plasmid-loaded PEI-CD/Ad-Dox were tested in an *in vitro* retention experiment on SKOV-3 ovarian tumors, with PEI-CD/Ad-Dox/pTRAIL exhibiting the highest levels of cytotoxicity. When different groups were compared, more apoptotic cells and more significant expression of apoptotic proteins were found in the SNP-treated group^[155]^.

#### Lipid-based nanoparticles

The TRAIL gene carrying cationic nanoliposomes (pDsRedl-Cl-TRAIL/Lipofectamine 2000) transfected into dendritic cells induced apoptosis in LoVo colorectal cancerous cells *in vitro* and *in vivo*^[156]^. The synthesis of a tumor-targeted lipid/calcium/phosphate/protamine (LCPP) nanoparticle to deliver TRAIL pDNA into hepatocellular carcinoma (HCC) cells with an HCC-targeting peptide (SP94) gave rise to apoptosis. Due to the stimulation of calmodulin-dependent protein kinase II, SP1, and calcium signaling, SP94-LCPP NPs carrying TRAIL pDNA dramatically enhanced TRAIL expression and triggered TRAIL-mediated cytotoxicity in human (Hep3B, JHH-7) and murine (HCA-1) HCC cells. Compared to previous treatments, TRAIL pDNA supplied by SP94-LCPP NPs dramatically boosted the number of apoptotic cells in orthotopic HCA-1 tumors. Compared to the single treatments, the combination of sorafenib with NP-induced TRAIL production caused HCC cells to undergo significantly higher levels of apoptosis^[157]^.

A novel lipid (1, 2-di-(9Z-octadecenoyl)-3-biguanide-propane (DOBP)) was encapsulated with TNF-related apoptosis-inducing ligand plasmids (TRAIL plasmids) into lipid-protamine-DNA nanoparticles (LPD NPs) for systemic gene delivery. Comparing 1, 2-di-(9Z-octadecenoyl)-3-trimethylammonium-propane (DOTAP)-LPD-TRAIL nanoparticles to DOBP-LPD TRAIL nanoparticles, it was clear that the latter were significantly less effective at slowing tumor growth. In contrast to phosphate-buffered saline and Lipo-DOTAP, tumor tissues treated with metformin and Lipo-DOBP showed significantly greater levels of AMPK-alpha phosphorylation, while mTOR phosphorylation was downregulated^[158]^.

The synthesis of another type of nanoparticles (rHDL/PEI-LA/pTRAIL) comprising a reconstituted high-density lipoprotein (rHDL), lauric acid-coupled polyethyleneimine (PEI-LA) as an amphipathic positively-charged polymer with pTRAIL was engineered into mesenchymal stem cells (MSCs) for the treatment of lung metastasis of melanoma. *In vitro* and *in vivo*, the rHDL-mediated TRAIL-engineered MSCs had a promising potential to target the B16F10 pulmonary melanoma metastases^[159]^*.*

#### Albumin-based nanoparticles

TRAIL gene delivered with plasmid pORF-hTRAIL (pDNA), incorporated into cationic albumin-conjugated pegylated nanoparticles (CBSA-NP), was evaluated as a non-viral vector for gene therapy of gliomas; hTRAIL-mediated apoptosis was assessed by immunohistochemical analysis for active caspase-3 using an antibody that detected the cleaved p17 fragment at 14 days after intravenous administration of CBSA-NP-hTRAIL, which was confirmed by the presence of p17-positive tumor cells^[160]^.

## CONCLUSION

TRAIL is an apoptosis-inducing ligand that kills cancer cells without affecting normal cells. TRAIL binds to specific death receptors on the cell surface and triggers the apoptosis extrinsic and intrinsic pathways. The targeted killing of specific tumor cells makes it a promising molecule for cancer treatment. TRAIL receptor agonists designed to bind and activate death receptors in the absence of TRAIL proved promising. Interestingly, TRAIL failed in clinical trials due to its relatively short half-life, decreased protein stability, reduced bioavailability, and resistant mechanisms exhibited by cancer cells to evade apoptosis. For successful therapy, the bioavailability of TRAIL was enhanced by synthesizing soluble and stable peptides of TRAIL and encapsulating them in nano-formulations that aided the targeted delivery of stable, active TRAIL to tumor cells. Various organic, inorganic, and hybrid nanoparticles carrying the TRAIL protein, gene, or receptor agonists were tested in *in vitro* studies in several cancer cell lines. Nanoparticle-mediated TRAIL delivery significantly enhanced apoptosis by increasing death receptor clustering, DISC formation, and caspase activation. Combinatorial treatment of tumor cells with nano-TRAIL and other chemotherapeutic agents also sensitized the resistant cancer cells to TRAIL. These studies suggest that applying nanotechnology to TRAIL therapy is successful in cell lines and preclinical animal models. Further experimentation and standardization are required for the success of nano-formulations of TRAIL.

Insights are required to search for biomarkers that can mark cells as TRAIL-sensitive, novel methods to deliver TRAIL precisely, and the use of TRAIL combined with other medications/natural products to overcome resistance. One compelling approach is the application of TRAIL nanoparticles in cancer treatment for the delivery of TRAIL protein or gene to tumor cells.

## DECLARATION

### Acknowledgments

The authors acknowledge the use of Servier Medical Art as Figures 1-3 were partly generated using Servier Medical Art, provided by Servier, licensed under a Creative Commons Attribution 3.0 unported license.

### Authors’ contributions

Wrote and edited portions of the manuscript: Gampa SC, Garimella SV, Pandrangi SL

### Availability of data and materials

Not applicable.

### Financial support and sponsorship

Funding from the Department of Science and Technology, Govt of India (SERB-TAR_2018_001127), the University Grants Commission [FNo 30-456/2018(BSR)]; GITAM Research Seed Grant (2021/0036) to Dr. Garimella SV and fellowship to Gampa SC from GITAM (deemed to be University) are greatly acknowledged.

### Conflicts of interest

All authors declared that there are no conflicts of interest.

### Ethical approval and consent to participate

Not applicable.

### Consent for publication

Not applicable.

### Copyright

© The Author(s) 2023.
